# Retraction Note: Salt stress amelioration and nutrient strengthening in spinach (*Spinacia oleracea* L.) via biochar amendment and zinc fortification: seed priming versus foliar application

**DOI:** 10.1038/s41598-026-43112-8

**Published:** 2026-03-09

**Authors:** Shoaib Ahmad, Adiba Khan Sehrish, Afzal Hussain, Lidan Zhang, Sarah Owdah Alomrani, Azeem Ahmad, Khalid A. Al-Ghanim, Mohammad Ali Alshehri, Shafaqat Ali, Pallab K. Sarker

**Affiliations:** 1 State Key Laboratory of Pollution Control and Resource Reuse, School of the Environment, Nanjing University, Nanjing, 210023 Jiangsu China; 2 Department of Environmental Sciences, The University of Lahore, Lahore, 54590 Pakistan; 3 Department of Biology, College of Science and Arts, Najran University, 66252 Najran, Saudi Arabia; 4 Soil and Water Chemistry Laboratory, Institute of Soil and Environment Sciences, University of Agriculture, Faisalabad, Pakistan; 5 Department of Zoology, College of Science, King Saud University, 11451 Riyadh, Saudi Arabia; 6 Department of Biology, Faculty of Science, University of Tabuk, 71491 Tabuk, Saudi Arabia; 7 Department of Environmental Sciences, Government College University Faisalabad, Faisalabad, 38000 Pakistan; 8 Environmental Studies Department, University of California Santa Cruz, Santa Cruz, CA 95060 USA; 9 Department of Biological Sciences and Technology, China Medical University, Taichung, 40402 Taiwan

Retraction of: *Scientific Reports* 10.1038/s41598-024-65834-3, published online 01 July 2024

The Editors have retracted this Article.

Initial concerns were raised over the presence of erroneous values in Tables 1 and 2. Subsequent investigation uncovered inconsistencies in the Authors’ description of EDX provenance and dating of FTIR images. Additionally, the authorship of this paper could not be verified. Pallab K. Sarker has stated that he had played no role in the creation of this article. The Editors have therefore lost confidence in the integrity of the findings.

Shoaib Ahmad, Adiba Khan Sehrish, Afzal Hussain, Shafaqat Ali and Pallab K. Sarker did not explicitly state if they agree or disagree with this retraction. Lidan Zhang, Sarah Owdah Alomrani, Azeem Ahmad, Khalid A. Al-Ghanim, Mohammad Ali Alshehri did not reply to the correspondence from Editors about this retraction.

